# Surveillance prostate biopsy utilizing piflufolastat F 18-PET/CT targeting in the intact prostate – A case report

**DOI:** 10.1016/j.eucr.2023.102352

**Published:** 2023-02-14

**Authors:** Tim Grossman, Kathryn A. Morton

**Affiliations:** a St. Peters Health, Helena, MT, USA; b University of Utah School of Medicine, Salt Lake City, UT, USA

**Keywords:** Piflufolastat F 18, PET/CT, PSMA-Targeted imaging

## Abstract

75-year-old patient with cochlear implant diagnosed with very low risk prostate cancer (PSA 6.44 ng/mL, and Grade Group 1 (left apical core)) managed on Active Surveillance (AS). After four years monitoring on AS, PSA was observed to increase to 10.84 and patient was reevaluated for disease progression. Due to cochlear implant, multiparametric MRI was not an imaging option and patient was referred for piflufolastat F 18-PET/CT. In addition to previously identified left sided lesion, tracer uptake was observed within the posterior transition and peripheral zone of the right lobe of the prostate, which ultimately confirmed disease progression on targeted biopsy.

## 1 Introduction

^18^F-DCFPyL (F-18 piflufolastat F 18, trade name PYLARIFY) is a fluorinated small molecule PSMA PET probe specifically targeting PSMA, a protein expressed in high concentration in most prostate cancer cells.^1^ This radiotracer was FDA approved in May 2021 for PET imaging of high-risk primary and recurrent prostate cancer.^2^

## 2 Case presentation

Patient is 75 year old, hearing impaired man with a cochlear implant originally diagnosed by transrectal ultrasound (TRUS) and biopsy in April 2018 with very low risk prostate cancer, a PSA of 6.44 ng/ml, and Grade Group 1 (left apical core). Patient elected active surveillance (AS) and was followed by semiannual exams and PSA levels. Patient remained clinically stable until July 2021 when the PSA was reported as 7.34 ng/ml. He underwent surveillance TRUS with prostate biopsy, again demonstrating left apical very low risk disease.

Patient was noted to have PSA increase to 10.84 ng/ml in January 2022. Decision to reimage and screen for multifocal or discordant lesion was made. Patient was not a candidate for multiparametric MRI because of his cochlear implant, and was referred to Huntsman Cancer Institute for piflufolastat F 18-PET/CT.

In February 2022, he received 4.15 mL of piflufolastat F 18 at a concentration of 2.17 mCi/mL and then scanned from skull vertex to mid-thigh using a Siemens Biograph 64 Visions 600 PET camera, 64 slice. Total dose was 9.5 mCi with time from injection to imaging of 73 minutes. CT technique included iodinated-contrast enhanced sequences as well as CT of the chest. PET images were corrected for attenuation using CT transmission data.

**Fig. 1:**
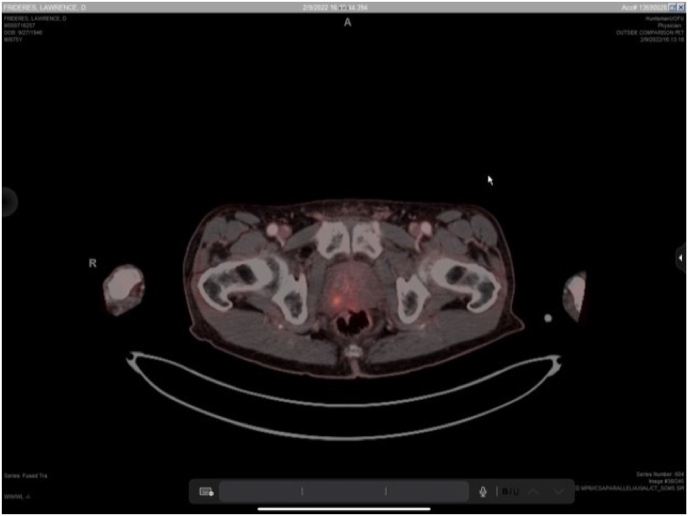
Focally intense area stereotactically mapped and biopsied at the junction of the right apical posterior transition and peripheral zones.

The study demonstrated focal right apical avidity (Fig. 1) within the posterior transition and peripheral zone of the prostate thought to represent an undiagnosed secondary lesion. There was also tracer uptake within the posterior transition and peripheral zone of the left lobe of the prostate, extending from mid (Fig. 2a) to apex (Fig. 2b). Known pathologic Gleason 3 + 3 disease was previously found in this area during his initial and surveillance biopsies and therefore served as an internal control.

**Fig. 2a:**
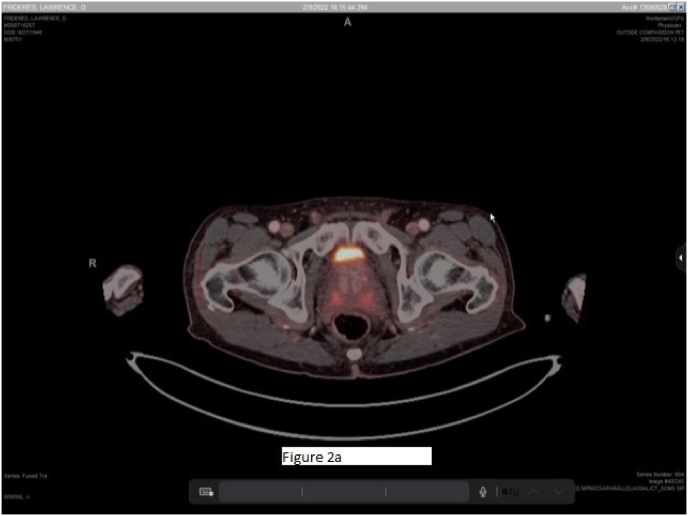
Cross sectional imaging in axial plane demonstrating bilateral tracer uptake in posterior transition and peripheral zones mid prostate. Both lesions extended apically but with less symmetry towards the intense metabolic activity seen in Fig. 1.

**Fig. 2b:**
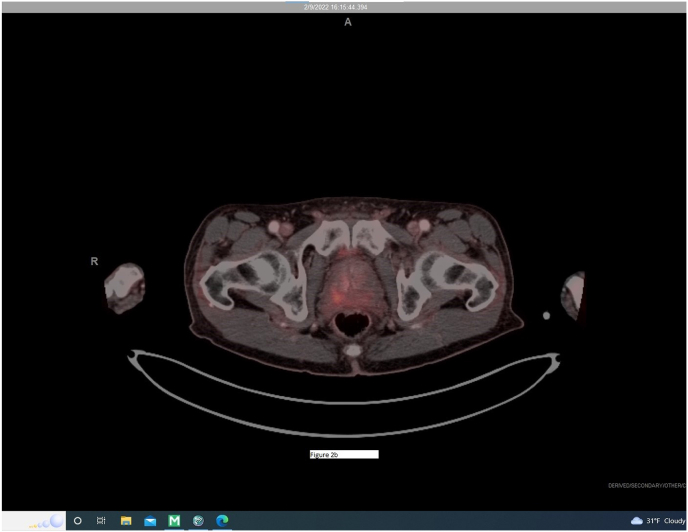
Bilateral apical extension of metabolic activity at junction of transitional and peripheral zones.

The 5 mm lesion in the right apex at the junction between the transition and peripheral zone (Fig. 3a) was successfully targeted utilizing stereotactic techniques and a CIVCO Micro-Touch Brachytherapy Stepper Unit (Fig. 3b). This demonstrated grade group 2 adenocarcinoma consistent with clinical progression.

**Fig. 3a:**
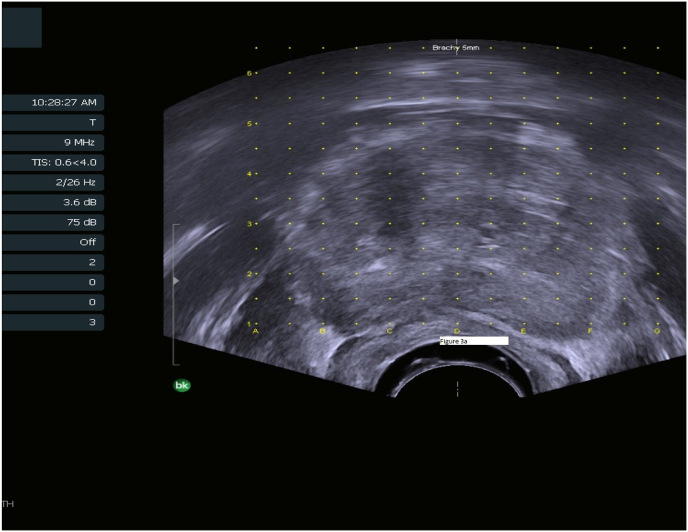
Intraoperative view of area corresponding to Fig. 1. This demonstrates subtle splaying of junctional calcification at coordinate b 2.0–2.5 which corresponded to targeted epicenter.

**Fig. 3b:**
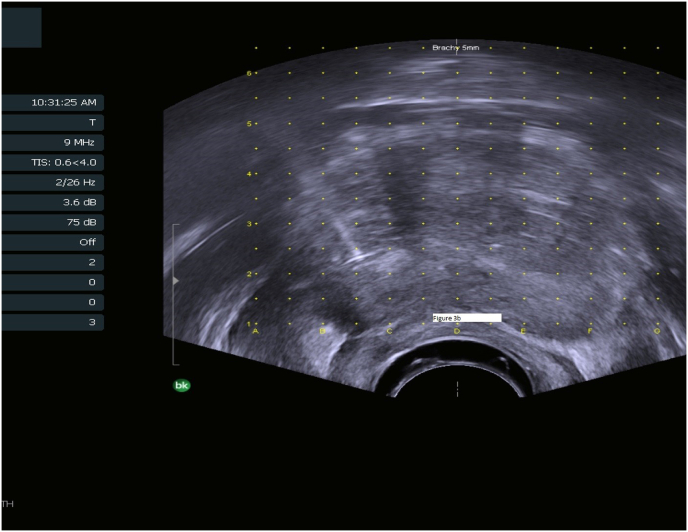
Intraoperative biopsy procedure.

## 3 Discussion

To our knowledge, this represents the first reported successful use of piflufolastat F18-PET/CT localization for targeted biopsy as a part of active surveillance monitoring for very low risk prostate cancer.

## 4 Conclusion

PSMA targeting imaging is expected to cause a paradigm shift in the current workup and management of prostate cancer patients with suspected metastasis or recurrent disease. This case demonstrates the added value, precision and utility that PSMA imaging may provide in the follow up and management of men on AS by localizing areas of suspected disease within the prostate itself.

## Funding

This research did not receive any specific grant from funding agencies in the public, commercial, or not-for-profit sectors.

## Declaration of competing interest

None.
